# *catena*-Poly[[(isoquinoline-κ*N*)(triphenylphospane-κ*P*)copper(I)]-μ-thio­cyanato-κ^2^ *N*:*S*]

**DOI:** 10.1107/S1600536812004837

**Published:** 2012-02-10

**Authors:** Jian-Bao Li, Li-Li Zhou, Man-Hua Wu, Qiong-Hua Jin, Cun-Lin Zhang

**Affiliations:** a Department of Chemistry, Capital Normal University, Beijing 100048, People’s Republic of China; b Research Center for Import-Export Chemicals Safety of the General Administration of Quality Supervision, Inspection and Quarantine of the People’s Republic of China (AQSIQ), Beijing 100123, People’s Republic of China; c Key Laboratory of Terahertz Optoelectronics of the Ministry of Education, Department of Physics, Capital Normal University, Beijing 100048, People’s Republic of China

## Abstract

In the title coordination compound, [Cu(NCS)(C_9_H_7_N)(C_18_H_15_P)]_*n*_, the Cu^I^ atom is tetra­hedrally coordinated by one N atom from an isoquinoline ligand, one P atom from a triphenyl­phospane ligand, and one N and one S atom from two thio­cyanate anions. The thio­cyanide anions bridge the Cu^I^ atoms into a chain along [100]. π–π inter­actions between the pyridine and benzene rings of the isoquinoline ligands connect the chains [centroid-to-centroid distance = 3.722 (3) Å].

## Related literature

For background to the applications of copper(I) complexes, see: Dai *et al.* (2010 ▶); Jin *et al.* (2010 ▶); Lu *et al.* (1997 ▶); Song *et al.* (2010 ▶). For related structures, see: Jin *et al.* (1999 ▶); Li, Wu *et al.* (2011 ▶); Li, Xiao *et al.* (2011 ▶).
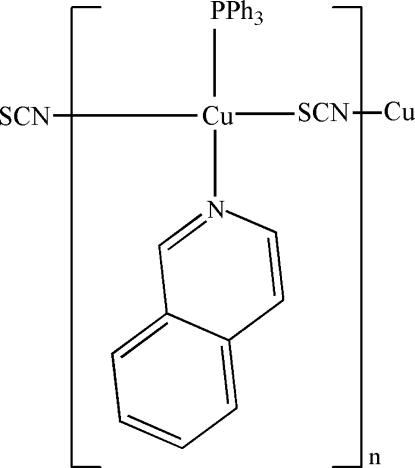


## Experimental

### 

#### Crystal data

[Cu(NCS)(C_9_H_7_N)(C_18_H_15_P)]
*M* *_r_* = 513.05
Monoclinic, 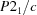

*a* = 12.9573 (13) Å
*b* = 10.5506 (11) Å
*c* = 18.9241 (18) Å
β = 108.961 (1)°
*V* = 2446.7 (4) Å^3^
*Z* = 4
Mo *K*α radiation
μ = 1.06 mm^−1^
*T* = 298 K
0.32 × 0.21 × 0.19 mm

#### Data collection

Bruker SMART 1000 CCD diffractometer
Absorption correction: multi-scan (*SADABS*; Bruker, 2001 ▶) *T*_min_ = 0.727, *T*_max_ = 0.824
12067 measured reflections
4313 independent reflections
2656 reflections with *I* > 2σ(*I*)
*R*_int_ = 0.042

#### Refinement

*R*[*F* ^2^ > 2σ(*F* ^2^)] = 0.041
*wR*(*F* ^2^) = 0.108
*S* = 1.05
4313 reflections
298 parameters
H-atom parameters constrained
Δρ_max_ = 0.41 e Å^−3^
Δρ_min_ = −0.25 e Å^−3^

### 

Data collection: *SMART* (Bruker, 2007 ▶); cell refinement: *SAINT-Plus* (Bruker, 2007 ▶); data reduction: *SAINT-Plus*; program(s) used to solve structure: *SHELXS97* (Sheldrick, 2008 ▶); program(s) used to refine structure: *SHELXL97* (Sheldrick, 2008 ▶); molecular graphics: *DIAMOND* (Brandenburg, 1999 ▶); software used to prepare material for publication: *SHELXTL* (Sheldrick, 2008 ▶).

## Supplementary Material

Crystal structure: contains datablock(s) global, I. DOI: 10.1107/S1600536812004837/hy2511sup1.cif

Structure factors: contains datablock(s) I. DOI: 10.1107/S1600536812004837/hy2511Isup2.hkl

Additional supplementary materials:  crystallographic information; 3D view; checkCIF report

## Figures and Tables

**Table 1:** Selected bond lengths (Å)

Cu1—N1	2.087 (3)
Cu1—N2^i^	1.991 (4)
Cu1—P1	2.2282 (11)
Cu1—S1	2.3781 (12)

Symmetry code: (i) 
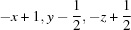
.

**Fig. 1.**
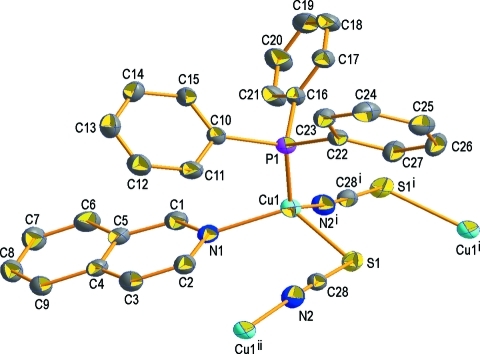
The asymmetric unit of the title complex. Displacement ellipsoids are shown at the 30% probability level. H atoms are omitted for clarity. [Symmetry codes: (i) 1 - x, -1/2 + y, 1/2 - z; (ii) 1 - x, 1/2 + y, 1/2 - z.]

**Fig. 2.**
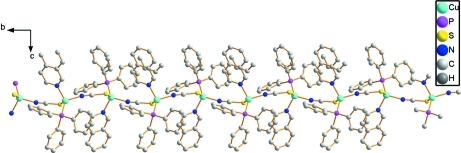
A view of the chain structure in the title compound.

## supplementary crystallographic information

### Comment

Many research efforts have been devoted to copper(I) complexes due to their interesting coordination chemistry and potential applications in photography, electrochemical processes, antimicrobial and antitumor activities (Dai *et al.*, 2010; Jin *et al.*, 2010; Lu *et al.*, 1997; Song *et al.*, 2010). Recently, we obtained some copper(I) complexes containing phosphine and isoquinoline (iq) ligands and coordinated anions (Li, Wu *et al.*, 2011; Li, Xiao *et al.*, 2011). Continuing these efforts, we report here the title compound, (I).

The compound was synthesized by the reaction of copper(I) salt with triphenylphospane (PPh_3_) and iq in a mixed solution of dichloromethane and methanol. The molar ratio of Cu(I):PPh_3_ (1:1) and the excess of iq are very important for the generation of this compound. The excess of iq facilitates its coordination to Cu(I) atom because the coordination ability of iq is weaker than that of PPh_3_ and SCN^-^ anion.

The Cu^I^ atom is bonded to one N atom from an iq ligand, one P atom from a PPh_3_ ligand, one S and one N atom from two SCN^-^ anions (Fig. 1). The SCN^-^ anion behaves as a bridging ligand. The structure of the title compound is similar to that of [CuBr(PPh_3_)_2_(iq)] (II) (Li, Wu *et al.*, 2011), [CuCl(PPh_3_)(iq)]_2_, (III) (Li, Xiao *et al.*, 2011), and [CuI(PPh_3_)(quinoline)]_2_ (IV) (Jin *et al.*, 1999). The bond length Cu1—P1 [2.2282 (11) Å] (Table 1) is shorter than the corresponding distances in (II) [2.2789 (14) Å] and (IV) [2.2466 (11) Å]. The Cu1—N1 bond length [2.087 (3) Å] is also shorter than the corresponding values in (II) [2.097 (3) Å] and (IV) [2.135 (4) Å]. The Cu1—P1 [2.1945 (8) Å] and Cu1—N1 [2.066 (2) Å] in (III) are shorter than the corresponding values in (I), (II) and (IV). The bond angle N1—Cu1—P1 [110.38 (9)°] in (I) is smaller than those in (III) [120.23 (7)°] and (IV) [117.20 (8)°] but larger than that in (II) [101.51 (12)°], while the bond angle P1—Cu1—S1 [113.85 (4)°] is smaller than those in (II) [115.88 (4)°], (III) [116.34 (3)°] and (IV) [114.70 (3)°]. The thiocyanide anions bridge the Cu^I^ atoms into a chain along [100] (Fig. 2). π–π interactions between the pyridine and benzene rings of the iq ligands connect the chains [centroid–centroid distance = 3.722 (3) Å].

### Experimental

The title complex was prepared by adding PPh_3_ (0.3 mmol, 0.079 g) into a mixture of CH_2_Cl_2_ (5 ml) and MeOH (5 ml) containing CuSCN (0.3 mmol, 0.036 g) and excess iq. The stirring continued for 3 h. After slow evaporation of the filtrate at ambient temperature for several days, yellow strip-shaped crystals were obtained. Crystals suitable for single-crystal X-ray diffraction were selected directly from the sample as prepared.

### Refinement

H atoms were positioned geometrically and refined as riding atoms, with C—H = 0.93 Å and with *U*_iso_(H) = 1.2*U*_eq_(C).

### Crystal data

**Table:**

[Cu(NCS)(C_9_H_7_N)(C_18_H_15_P)]	*F*(000) = 1056
*M**_r_* = 513.05	*D*_x_ = 1.393 Mg m^−^^3^
Monoclinic, *P*2_1_/*c*	Mo *K*α radiation, λ = 0.71073 Å
Hall symbol: -P 2ybc	Cell parameters from 2854 reflections
*a* = 12.9573 (13) Å	θ = 2.6–22.5°
*b* = 10.5506 (11) Å	µ = 1.06 mm^−^^1^
*c* = 18.9241 (18) Å	*T* = 298 K
β = 108.961 (1)°	Prism, yellow
*V* = 2446.7 (4) Å^3^	0.32 × 0.21 × 0.19 mm
*Z* = 4	

### Data collection

**Table:**

Bruker SMART 1000 CCD diffractometer	4313 independent reflections
Radiation source: fine-focus sealed tube	2656 reflections with *I* > 2σ(*I*)
Graphite monochromator	*R*_int_ = 0.042
φ and ω scans	θ_max_ = 25.0°, θ_min_ = 2.6°
Absorption correction: multi-scan (*SADABS*; Bruker, 2001)	*h* = −15→15
*T*_min_ = 0.727, *T*_max_ = 0.824	*k* = −12→12
12067 measured reflections	*l* = −22→18

### Refinement

**Table:**

Refinement on *F*^2^	Primary atom site location: structure-invariant direct methods
Least-squares matrix: full	Secondary atom site location: difference Fourier map
*R*[*F*^2^ > 2σ(*F*^2^)] = 0.041	Hydrogen site location: inferred from neighbouring sites
*wR*(*F*^2^) = 0.108	H-atom parameters constrained
*S* = 1.05	*w* = 1/[σ^2^(*F*_o_^2^) + (0.0387*P*)^2^ + 1.2684*P*] where *P* = (*F*_o_^2^ + 2*F*_c_^2^)/3
4313 reflections	(Δ/σ)_max_ < 0.001
298 parameters	Δρ_max_ = 0.41 e Å^−^^3^
0 restraints	Δρ_min_ = −0.25 e Å^−^^3^

### Special details

**Table:**

Geometry. All e.s.d.'s (except the e.s.d. in the dihedral angle between two l.s. planes) are estimated using the full covariance matrix. The cell e.s.d.'s are taken into account individually in the estimation of e.s.d.'s in distances, angles and torsion angles; correlations between e.s.d.'s in cell parameters are only used when they are defined by crystal symmetry. An approximate (isotropic) treatment of cell e.s.d.'s is used for estimating e.s.d.'s involving l.s. planes.
Refinement. Refinement of *F*^2^ against ALL reflections. The weighted *R*-factor *wR* and goodness of fit *S* are based on *F*^2^, conventional *R*-factors *R* are based on *F*, with *F* set to zero for negative *F*^2^. The threshold expression of *F*^2^ > σ(*F*^2^) is used only for calculating *R*-factors(gt) *etc*. and is not relevant to the choice of reflections for refinement. *R*-factors based on *F*^2^ are statistically about twice as large as those based on *F*, and *R*-factors based on ALL data will be even larger.

### Fractional atomic coordinates and isotropic or equivalent isotropic displacement parameters (Å^2^)

**Table:**

	*x*	*y*	*z*	*U*_iso_*/*U*_eq_	
Cu1	0.60682 (4)	0.39666 (4)	0.25965 (3)	0.04929 (17)	
P1	0.75772 (8)	0.43807 (9)	0.35473 (6)	0.0449 (3)	
S1	0.44293 (9)	0.45262 (10)	0.28281 (7)	0.0572 (3)	
N1	0.6132 (3)	0.4852 (3)	0.16249 (18)	0.0523 (9)	
N2	0.4224 (3)	0.7158 (3)	0.2701 (2)	0.0638 (10)	
C1	0.6628 (3)	0.4305 (4)	0.1208 (2)	0.0575 (11)	
H1	0.6942	0.3515	0.1357	0.069*	
C2	0.5675 (3)	0.6006 (4)	0.1397 (2)	0.0581 (11)	
H2	0.5312	0.6408	0.1685	0.070*	
C3	0.5719 (4)	0.6601 (4)	0.0774 (3)	0.0629 (12)	
H3	0.5406	0.7398	0.0650	0.076*	
C4	0.6232 (3)	0.6021 (4)	0.0322 (2)	0.0499 (10)	
C5	0.6721 (3)	0.4842 (4)	0.0535 (2)	0.0486 (10)	
C6	0.7248 (4)	0.4218 (5)	0.0107 (3)	0.0700 (13)	
H6	0.7581	0.3440	0.0262	0.084*	
C7	0.7275 (4)	0.4745 (5)	−0.0540 (3)	0.0770 (14)	
H7	0.7627	0.4329	−0.0830	0.092*	
C8	0.6774 (4)	0.5913 (5)	−0.0770 (3)	0.0744 (14)	
H8	0.6794	0.6257	−0.1218	0.089*	
C9	0.6265 (4)	0.6557 (5)	−0.0369 (3)	0.0653 (12)	
H9	0.5939	0.7335	−0.0535	0.078*	
C10	0.8397 (3)	0.5678 (3)	0.3374 (2)	0.0437 (9)	
C11	0.7862 (3)	0.6785 (4)	0.3058 (2)	0.0566 (11)	
H11	0.7108	0.6840	0.2942	0.068*	
C12	0.8432 (4)	0.7799 (4)	0.2916 (3)	0.0689 (13)	
H12	0.8066	0.8541	0.2716	0.083*	
C13	0.9535 (4)	0.7718 (4)	0.3067 (3)	0.0694 (13)	
H13	0.9915	0.8398	0.2959	0.083*	
C14	1.0081 (4)	0.6638 (4)	0.3376 (2)	0.0641 (12)	
H14	1.0833	0.6588	0.3486	0.077*	
C15	0.9514 (3)	0.5630 (4)	0.3524 (2)	0.0546 (11)	
H15	0.9891	0.4897	0.3731	0.065*	
C16	0.8556 (3)	0.3077 (3)	0.3805 (2)	0.0475 (10)	
C17	0.9200 (3)	0.2820 (4)	0.4534 (2)	0.0564 (11)	
H17	0.9139	0.3318	0.4924	0.068*	
C18	0.9935 (4)	0.1819 (4)	0.4680 (3)	0.0672 (13)	
H18	1.0364	0.1647	0.5170	0.081*	
C19	1.0033 (4)	0.1087 (4)	0.4113 (3)	0.0755 (15)	
H19	1.0534	0.0425	0.4216	0.091*	
C20	0.9398 (4)	0.1320 (4)	0.3393 (3)	0.0807 (15)	
H20	0.9462	0.0814	0.3007	0.097*	
C21	0.8663 (4)	0.2305 (4)	0.3241 (3)	0.0645 (12)	
H21	0.8230	0.2456	0.2750	0.077*	
C22	0.7412 (3)	0.4807 (4)	0.4436 (2)	0.0457 (10)	
C23	0.8026 (3)	0.5724 (4)	0.4911 (2)	0.0563 (11)	
H23	0.8544	0.6179	0.4772	0.068*	
C24	0.7881 (4)	0.5973 (4)	0.5587 (2)	0.0674 (13)	
H24	0.8301	0.6592	0.5900	0.081*	
C25	0.7125 (4)	0.5319 (5)	0.5801 (3)	0.0747 (14)	
H25	0.7030	0.5491	0.6258	0.090*	
C26	0.6510 (4)	0.4414 (5)	0.5342 (3)	0.0824 (15)	
H26	0.6001	0.3957	0.5489	0.099*	
C27	0.6640 (4)	0.4169 (4)	0.4657 (3)	0.0682 (13)	
H27	0.6200	0.3565	0.4342	0.082*	
C28	0.4320 (3)	0.6079 (4)	0.2756 (2)	0.0466 (10)	

### Atomic displacement parameters (Å^2^)

**Table:**

	*U*^11^	*U*^22^	*U*^33^	*U*^12^	*U*^13^	*U*^23^
Cu1	0.0578 (3)	0.0447 (3)	0.0480 (3)	−0.0041 (2)	0.0207 (2)	−0.0004 (2)
P1	0.0494 (6)	0.0434 (6)	0.0444 (6)	−0.0001 (5)	0.0187 (5)	0.0021 (5)
S1	0.0612 (7)	0.0445 (6)	0.0755 (8)	0.0004 (5)	0.0353 (6)	0.0060 (5)
N1	0.051 (2)	0.061 (2)	0.048 (2)	−0.0058 (17)	0.0210 (18)	−0.0031 (17)
N2	0.076 (3)	0.050 (2)	0.073 (3)	−0.0087 (19)	0.035 (2)	−0.0070 (19)
C1	0.057 (3)	0.058 (3)	0.057 (3)	−0.003 (2)	0.019 (2)	−0.002 (2)
C2	0.061 (3)	0.060 (3)	0.062 (3)	0.003 (2)	0.033 (2)	0.002 (2)
C3	0.071 (3)	0.055 (3)	0.072 (3)	0.007 (2)	0.035 (3)	0.006 (2)
C4	0.042 (2)	0.057 (3)	0.050 (3)	−0.008 (2)	0.016 (2)	−0.007 (2)
C5	0.043 (2)	0.054 (3)	0.051 (3)	−0.0049 (19)	0.018 (2)	−0.007 (2)
C6	0.069 (3)	0.077 (3)	0.070 (3)	0.009 (2)	0.031 (3)	−0.004 (3)
C7	0.089 (4)	0.094 (4)	0.058 (3)	−0.005 (3)	0.037 (3)	−0.004 (3)
C8	0.081 (3)	0.101 (4)	0.047 (3)	−0.014 (3)	0.027 (3)	0.003 (3)
C9	0.066 (3)	0.075 (3)	0.056 (3)	−0.009 (2)	0.021 (3)	0.009 (2)
C10	0.053 (2)	0.043 (2)	0.038 (2)	−0.0006 (18)	0.020 (2)	−0.0005 (17)
C11	0.054 (3)	0.053 (3)	0.061 (3)	0.000 (2)	0.016 (2)	0.003 (2)
C12	0.084 (4)	0.047 (3)	0.081 (4)	0.006 (2)	0.034 (3)	0.014 (2)
C13	0.087 (4)	0.053 (3)	0.080 (4)	−0.014 (3)	0.043 (3)	0.005 (2)
C14	0.061 (3)	0.066 (3)	0.075 (3)	−0.007 (2)	0.035 (3)	0.005 (3)
C15	0.059 (3)	0.053 (3)	0.060 (3)	0.004 (2)	0.031 (2)	0.010 (2)
C16	0.056 (3)	0.041 (2)	0.050 (3)	−0.0028 (19)	0.024 (2)	0.0048 (19)
C17	0.061 (3)	0.051 (3)	0.057 (3)	−0.003 (2)	0.019 (2)	0.005 (2)
C18	0.066 (3)	0.058 (3)	0.072 (3)	0.004 (2)	0.013 (3)	0.023 (3)
C19	0.076 (3)	0.053 (3)	0.105 (5)	0.019 (2)	0.039 (3)	0.023 (3)
C20	0.108 (4)	0.063 (3)	0.082 (4)	0.027 (3)	0.046 (3)	0.013 (3)
C21	0.089 (3)	0.055 (3)	0.055 (3)	0.017 (2)	0.030 (3)	0.012 (2)
C22	0.046 (2)	0.050 (2)	0.043 (2)	0.0034 (19)	0.017 (2)	0.0042 (19)
C23	0.058 (3)	0.061 (3)	0.055 (3)	−0.001 (2)	0.025 (2)	−0.002 (2)
C24	0.068 (3)	0.081 (3)	0.054 (3)	0.006 (3)	0.021 (3)	−0.014 (2)
C25	0.069 (3)	0.111 (4)	0.054 (3)	0.016 (3)	0.033 (3)	0.000 (3)
C26	0.070 (3)	0.127 (5)	0.062 (3)	−0.021 (3)	0.037 (3)	0.002 (3)
C27	0.064 (3)	0.091 (4)	0.054 (3)	−0.020 (3)	0.025 (2)	−0.005 (2)
C28	0.049 (2)	0.052 (3)	0.042 (2)	−0.002 (2)	0.0204 (19)	0.002 (2)

### Geometric parameters (Å, º)

**Table:**

Cu1—N1	2.087 (3)	C11—H11	0.9300
Cu1—N2^i^	1.991 (4)	C12—C13	1.366 (6)
Cu1—P1	2.2282 (11)	C12—H12	0.9300
Cu1—S1	2.3781 (12)	C13—C14	1.369 (6)
P1—C22	1.820 (4)	C13—H13	0.9300
P1—C16	1.826 (4)	C14—C15	1.372 (5)
P1—C10	1.826 (4)	C14—H14	0.9300
S1—C28	1.647 (4)	C15—H15	0.9300
N1—C1	1.303 (5)	C16—C21	1.385 (5)
N1—C2	1.362 (5)	C16—C17	1.387 (5)
N2—C28	1.146 (4)	C17—C18	1.389 (6)
C1—C5	1.435 (6)	C17—H17	0.9300
C1—H1	0.9300	C18—C19	1.361 (6)
C2—C3	1.352 (6)	C18—H18	0.9300
C2—H2	0.9300	C19—C20	1.366 (6)
C3—C4	1.385 (5)	C19—H19	0.9300
C3—H3	0.9300	C20—C21	1.376 (6)
C4—C5	1.395 (5)	C20—H20	0.9300
C4—C9	1.438 (6)	C21—H21	0.9300
C5—C6	1.384 (6)	C22—C27	1.378 (5)
C6—C7	1.355 (6)	C22—C23	1.383 (5)
C6—H6	0.9300	C23—C24	1.378 (6)
C7—C8	1.394 (6)	C23—H23	0.9300
C7—H7	0.9300	C24—C25	1.361 (6)
C8—C9	1.340 (6)	C24—H24	0.9300
C8—H8	0.9300	C25—C26	1.361 (6)
C9—H9	0.9300	C25—H25	0.9300
C10—C15	1.382 (5)	C26—C27	1.385 (6)
C10—C11	1.390 (5)	C26—H26	0.9300
C11—C12	1.376 (6)	C27—H27	0.9300
			
N2^i^—Cu1—N1	103.75 (14)	C13—C12—C11	120.2 (4)
N2^i^—Cu1—P1	116.93 (11)	C13—C12—H12	119.9
N1—Cu1—P1	110.38 (9)	C11—C12—H12	119.9
N2^i^—Cu1—S1	101.00 (11)	C12—C13—C14	120.1 (4)
N1—Cu1—S1	110.14 (10)	C12—C13—H13	120.0
P1—Cu1—S1	113.85 (4)	C14—C13—H13	120.0
C22—P1—C16	102.65 (18)	C13—C14—C15	119.8 (4)
C22—P1—C10	103.35 (17)	C13—C14—H14	120.1
C16—P1—C10	102.60 (17)	C15—C14—H14	120.1
C22—P1—Cu1	117.32 (13)	C14—C15—C10	121.6 (4)
C16—P1—Cu1	114.77 (13)	C14—C15—H15	119.2
C10—P1—Cu1	114.27 (13)	C10—C15—H15	119.2
C28—S1—Cu1	106.87 (14)	C21—C16—C17	118.2 (4)
C1—N1—C2	116.9 (4)	C21—C16—P1	118.2 (3)
C1—N1—Cu1	120.3 (3)	C17—C16—P1	123.7 (3)
C2—N1—Cu1	122.8 (3)	C16—C17—C18	120.0 (4)
N1—C1—C5	124.3 (4)	C16—C17—H17	120.0
N1—C1—H1	117.8	C18—C17—H17	120.0
C5—C1—H1	117.8	C19—C18—C17	120.5 (4)
C3—C2—N1	123.6 (4)	C19—C18—H18	119.7
C3—C2—H2	118.2	C17—C18—H18	119.7
N1—C2—H2	118.2	C18—C19—C20	120.3 (4)
C2—C3—C4	119.9 (4)	C18—C19—H19	119.8
C2—C3—H3	120.0	C20—C19—H19	119.8
C4—C3—H3	120.0	C19—C20—C21	119.7 (5)
C3—C4—C5	118.6 (4)	C19—C20—H20	120.1
C3—C4—C9	123.4 (4)	C21—C20—H20	120.1
C5—C4—C9	117.9 (4)	C20—C21—C16	121.3 (4)
C6—C5—C4	121.3 (4)	C20—C21—H21	119.3
C6—C5—C1	122.2 (4)	C16—C21—H21	119.3
C4—C5—C1	116.5 (4)	C27—C22—C23	117.8 (4)
C7—C6—C5	119.7 (5)	C27—C22—P1	118.3 (3)
C7—C6—H6	120.1	C23—C22—P1	123.9 (3)
C5—C6—H6	120.1	C24—C23—C22	120.9 (4)
C6—C7—C8	119.9 (5)	C24—C23—H23	119.6
C6—C7—H7	120.1	C22—C23—H23	119.6
C8—C7—H7	120.1	C25—C24—C23	120.5 (5)
C9—C8—C7	122.4 (5)	C25—C24—H24	119.8
C9—C8—H8	118.8	C23—C24—H24	119.8
C7—C8—H8	118.8	C26—C25—C24	119.7 (5)
C8—C9—C4	118.7 (4)	C26—C25—H25	120.2
C8—C9—H9	120.6	C24—C25—H25	120.2
C4—C9—H9	120.6	C25—C26—C27	120.3 (5)
C15—C10—C11	117.5 (4)	C25—C26—H26	119.9
C15—C10—P1	124.6 (3)	C27—C26—H26	119.9
C11—C10—P1	117.9 (3)	C22—C27—C26	120.9 (4)
C12—C11—C10	120.8 (4)	C22—C27—H27	119.6
C12—C11—H11	119.6	C26—C27—H27	119.6
C10—C11—H11	119.6	N2—C28—S1	178.8 (4)
			
N2^i^—Cu1—P1—C22	−106.25 (18)	Cu1—P1—C10—C15	−132.9 (3)
N1—Cu1—P1—C22	135.50 (18)	C22—P1—C10—C11	−82.6 (3)
S1—Cu1—P1—C22	11.04 (15)	C16—P1—C10—C11	170.9 (3)
N2^i^—Cu1—P1—C16	14.40 (19)	Cu1—P1—C10—C11	46.0 (3)
N1—Cu1—P1—C16	−103.84 (18)	C15—C10—C11—C12	−1.2 (6)
S1—Cu1—P1—C16	131.69 (14)	P1—C10—C11—C12	179.8 (3)
N2^i^—Cu1—P1—C10	132.54 (18)	C10—C11—C12—C13	1.7 (7)
N1—Cu1—P1—C10	14.30 (17)	C11—C12—C13—C14	−1.5 (7)
S1—Cu1—P1—C10	−110.16 (14)	C12—C13—C14—C15	1.0 (7)
N2^i^—Cu1—S1—C28	−158.11 (19)	C13—C14—C15—C10	−0.6 (7)
N1—Cu1—S1—C28	−48.89 (18)	C11—C10—C15—C14	0.7 (6)
P1—Cu1—S1—C28	75.70 (15)	P1—C10—C15—C14	179.6 (3)
N2^i^—Cu1—N1—C1	−41.4 (3)	C22—P1—C16—C21	163.7 (3)
P1—Cu1—N1—C1	84.6 (3)	C10—P1—C16—C21	−89.3 (3)
S1—Cu1—N1—C1	−148.8 (3)	Cu1—P1—C16—C21	35.3 (4)
N2^i^—Cu1—N1—C2	138.9 (3)	C22—P1—C16—C17	−16.5 (4)
P1—Cu1—N1—C2	−95.0 (3)	C10—P1—C16—C17	90.5 (4)
S1—Cu1—N1—C2	31.5 (3)	Cu1—P1—C16—C17	−145.0 (3)
C2—N1—C1—C5	0.2 (6)	C21—C16—C17—C18	0.7 (6)
Cu1—N1—C1—C5	−179.5 (3)	P1—C16—C17—C18	−179.1 (3)
C1—N1—C2—C3	−0.5 (6)	C16—C17—C18—C19	0.2 (6)
Cu1—N1—C2—C3	179.1 (3)	C17—C18—C19—C20	−0.8 (7)
N1—C2—C3—C4	1.4 (7)	C18—C19—C20—C21	0.5 (8)
C2—C3—C4—C5	−1.9 (6)	C19—C20—C21—C16	0.4 (7)
C2—C3—C4—C9	176.6 (4)	C17—C16—C21—C20	−0.9 (6)
C3—C4—C5—C6	−179.9 (4)	P1—C16—C21—C20	178.8 (4)
C9—C4—C5—C6	1.6 (6)	C16—P1—C22—C27	−87.1 (3)
C3—C4—C5—C1	1.5 (5)	C10—P1—C22—C27	166.4 (3)
C9—C4—C5—C1	−177.0 (3)	Cu1—P1—C22—C27	39.7 (4)
N1—C1—C5—C6	−179.3 (4)	C16—P1—C22—C23	92.0 (3)
N1—C1—C5—C4	−0.7 (6)	C10—P1—C22—C23	−14.5 (4)
C4—C5—C6—C7	−1.2 (7)	Cu1—P1—C22—C23	−141.2 (3)
C1—C5—C6—C7	177.4 (4)	C27—C22—C23—C24	1.1 (6)
C5—C6—C7—C8	0.0 (7)	P1—C22—C23—C24	−178.0 (3)
C6—C7—C8—C9	0.6 (8)	C22—C23—C24—C25	−0.1 (7)
C7—C8—C9—C4	−0.2 (7)	C23—C24—C25—C26	0.0 (7)
C3—C4—C9—C8	−179.4 (4)	C24—C25—C26—C27	−0.9 (8)
C5—C4—C9—C8	−1.0 (6)	C23—C22—C27—C26	−2.0 (6)
C22—P1—C10—C15	98.4 (4)	P1—C22—C27—C26	177.2 (4)
C16—P1—C10—C15	−8.1 (4)	C25—C26—C27—C22	1.9 (8)

Symmetry code: (i) −*x*+1, *y*−1/2, −*z*+1/2.
